# Shear Stress Modulates Osteoblast Cell and Nucleus Morphology and Volume

**DOI:** 10.3390/ijms21218361

**Published:** 2020-11-07

**Authors:** Jianfeng Jin, Richard T. Jaspers, Gang Wu, Joannes A.M. Korfage, Jenneke Klein-Nulend, Astrid D. Bakker

**Affiliations:** 1 Department of Oral Cell Biology, Academic Centre for Dentistry Amsterdam (ACTA), University of Amsterdam and Vrije Universiteit Amsterdam, Amsterdam Movement Sciences, 1081 LA Amsterdam, The Netherlands; j.jin@acta.nl (J.J.); j.kleinnulend@acta.nl (J.K.-N.); 2 Laboratory for Myology, Faculty of Behavioral and Movement Sciences, Vrije Universiteit Amsterdam, Amsterdam Movement Sciences, 1081 HZ Amsterdam, The Netherlands; r.t.jaspers@vu.nl; 3 Department of Oral Implantology and Prosthetic Dentistry, Academic Centre for Dentistry Amsterdam (ACTA), University of Amsterdam and Vrije Universiteit Amsterdam, Amsterdam Movement Sciences, 1081 LA Amsterdam, The Netherlands; g.wu@acta.nl; 4 Department of Oral and Maxillofacial Surgery/Oral Pathology, Amsterdam University Medical Centers-location VUmc and Academic Centre for Dentistry Amsterdam (ACTA), Amsterdam Movement Sciences, 1081 HV Amsterdam, The Netherlands; 5 Department of Functional Anatomy, Academic Centre for Dentistry Amsterdam (ACTA), University of Amsterdam and Vrije Universiteit Amsterdam, Amsterdam Movement Sciences, 1081 LA Amsterdam, The Netherlands; j.korfage@acta.nl

**Keywords:** bone, cell shape, cell signaling, cytoplasm volume, cytoskeleton, focal adhesions, mechanical stimulation, mechanotransduction, nucleus shape, pulsating fluid flow, three-dimensional imaging

## Abstract

Mechanical loading preserves bone mass and function—yet, little is known about the cell biological basis behind this preservation. For example, cell and nucleus morphology are critically important for cell function, but how these morphological characteristics are affected by the physiological mechanical loading of bone cells is under-investigated. This study aims to determine the effects of fluid shear stress on cell and nucleus morphology and volume of osteoblasts, and how these effects relate to changes in actin cytoskeleton and focal adhesion formation. Mouse calvaria 3T3-E1 (MC3T3-E1) osteoblasts were treated with or without 1 h pulsating fluid flow (PFF). Live-cell imaging was performed every 10 min during PFF and immediately after PFF. Cytoskeletal organization and focal adhesions were visualized, and gene and protein expression quantified. Two-dimensional (2D) and three-dimensional (3D) morphometric analyses were made using MeasureStack and medical imaging interaction toolkit (MITK) software. 2D-images revealed that 1 h PFF changed cell morphology from polygonal to triangular, and nucleus morphology from round to ellipsoid. PFF also reduced cell surface area (0.3-fold), cell volume (0.3-fold), and nucleus volume (0.2-fold). During PFF, the live-cell volume gradually decreased from 6000 to 3000 µm^3^. After PFF, α-tubulin orientation was more disorganized, but F-actin fluorescence intensity was enhanced, particularly around the nucleus. 3D-images obtained from Z-stacks indicated that PFF increased F-actin fluorescence signal distribution around the nucleus in the XZ and YZ direction (2.3-fold). PFF increased protein expression of phospho-paxillin (2.0-fold) and integrin-α5 (2.8-fold), but did not increase mRNA expression of paxillin-a (P*XNA*), paxillin-b (*PXNB*), integrin-α5 (*ITGA51*), or α-tubulin protein expression. In conclusion, PFF induced substantial changes in osteoblast cytoskeleton, as well as cell and nucleus morphology and volume, which was accompanied by elevated gene and protein expression of adhesion and structural proteins. More insights into the mechanisms whereby mechanical cues drive morphological changes in bone cells, and thereby, possibly in bone cell behavior, will aid the guidance of clinical treatment, particularly in the field of orthodontics, (oral) implantology, and orthopedics.

## 1. Introduction

In orthodontics, (oral) implantology, and orthopedics, mechanical stimuli are known to modulate bone mass, strength, and microstructure [1]. Changes in bone induced by orthodontic forces are modulated by the periodontal ligament [2]. However, direct sensing of mechanical loads, or lack thereof, by osteocytes and osteoblasts also plays an important role in the shaping of bone tissue [3,4,5]. Both osteocytes and osteoblasts are responsive to the loading-induced flow of fluid, albeit osteocytes are most responsive [6]. Since osteoblasts (which terminally differentiate into osteocytes) respond to fluid flow-induced shear stress in vitro, osteoblasts provide a practical model for studying bone cell responses to shear stress. Fluid flow induced by four-point-bending of rat tibiae in vivo has been confirmed by tracer methods [7]. After sensing mechanical signals, osteocytes or osteoblasts translate the mechanical signal into a biological response, through a process known as mechanotransduction.

Mechanotransduction plays a vital role in bone, where it eventually results in an anabolic or catabolic biological reaction. Pathological unloading of bone unequivocally leads to bone loss, while physiological mechanical stimuli decrease bone resorption and promote bone formation in vivo [8]. Further intensification of the mechanical stimulus leads to overloading-induced bone loss. Overloading causes microdamage, osteocyte apoptosis, and stimulation of osteoclast formation and activity, but it may also induce a direct catabolic response in bone cells [9]. It is not yet fully understood which factors determine whether a mechanical stimulus is osteo-anabolic or osteo-catabolic, or even how a physiological mechanical signal is altered into an anabolic chemical response within the cell. More knowledge of the mechanotransduction process would aid oral implantology, orthodontics, and orthopedics by increasing the understanding of the mechanisms driving mechanical loading-induced bone adaptation.

Mechanotransduction in bone can be divided into four distinct stages, i.e., mechanocoupling, biochemical coupling, the transmission of biochemical signals, and effector cell response [10]. Mechanocoupling, the transduction of mechanical energy, is executed by cells in mechanosensory molecular complexes. Biochemical coupling refers to the detection and conversion of mechanical stimuli into biochemical signals in the cell. One possible conversion mechanism is via the extracellular matrix-integrin-cytoskeletal axis [8]. Cells attach to their substrate through membrane-spanning glycoproteins (integrins). Integrin-α5 is of specific interest for osteoblasts and osteocytes, since it may open hemichannels and release prostaglandins in osteocytes treated by shear stress [11,12]. Moreover, integrin-α5 is required for osteoblast differentiation [13]. Integrins connect the extracellular matrix to the cytoskeleton, via several actin-associated proteins, e.g., α-actinin, talin, tensin, paxillin, and vinculin [14]. The signaling transduction adaptor paxillin activates cytoplasmic signals (e.g., Wnts) and directly changes the cytoskeletal structure via recombination or reorganization of microtubules and microfilaments [15]. Although not yet shown for osteoblasts, in many cell types, the cytoskeleton is connected to molecular complexes embedded in the nuclear envelope, thereby forming a continuous connection from the extracellular matrix all the way to the nucleus [16]. Therefore, mechanical stimuli can affect cell cytoskeletal structure and morphology, nuclear structure, nuclear shape, chromatin structure, and thereby perhaps, cell behavior [16,17,18,19,20,21]. After biochemical coupling, cells, such as osteocytes and osteoblasts can alter their activity, but also that of neighboring cells, via the production of signaling molecules [10,22,23].

Bone cell shape (e.g., round and flat) is closely related to cell function [24]. In vivo, osteoblasts undergo several stages of terminal differentiation, i.e., mature osteoblast, osteoblastic osteocyte, osteoid osteocyte, and mature osteocyte, each with their own function [25]. During these stages, a shift in cell volume distribution occurs, changing the osteoblast shape from cuboidal, via round to stellate or dendritic-shaped, resulting in 30% volume-reduction in the nascent osteocyte cell body, and 70% volume-reduction in the mature osteocyte cell body compared with the volume of an osteoblast cell body [25]. Interestingly, the nuclear shape also can be changed by mechanical stress. For example, hypo-osmotic stress increases the nuclear volume (round shape), whereas hyper-osmotic stress decreases nuclear volume (convoluted shape) [26]. Cell and nuclear volume and function may, thus, be related.

Little is known about the volume of cells of the osteoblast lineage and their nuclei in response to mechanical stimuli. Therefore, we aimed to investigate whether pulsating fluid flow (PFF) induces changes in three-dimensional (3D) cell and nucleus morphology. Moreover, the function of osteoblasts. We focused on the morphology (F-actin, paxillin, integrin-α5, and α-tubulin) and volume of cell and nucleus, since these can change by PFF-treatment. We analyzed the cellular depolymerization/polymerization status after mechanical loading by PFF based on changes in morphology and function (gene and protein expression) through the determination of (intracellular and extracellular) structures.

## 2. Results

### 2.1. 3D Cell and Nucleus Volume

**Figure 1:**
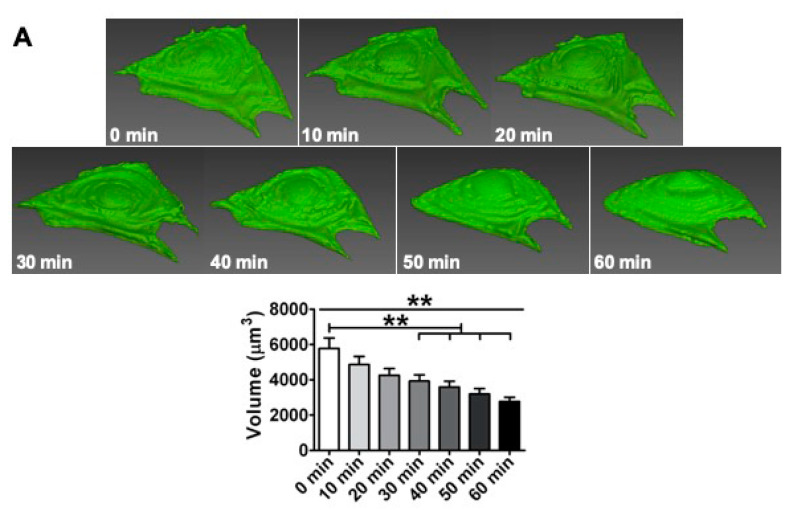

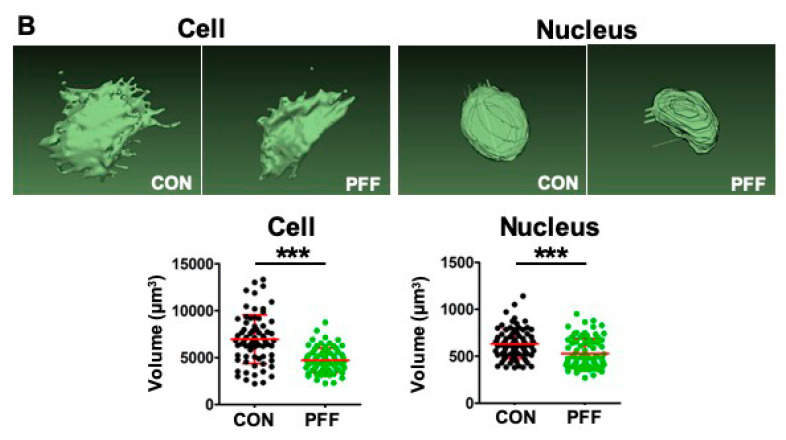
Effect of 1 h pulsating fluid flow (PFF) on live and fixed cell and nucleus volumes of MC3T3-E1 osteoblasts. (**A**) Volumetric reconstruction and cell volume of live cells exposed to PFF at different time points. *n* = 24 cells from six glass slides. (**B**) Volumetric reconstruction and volume of cells and nuclei in formaldehyde-fixed cells subjected to 1 h static control culture or PFF treatment. *n* = 70 cells from 9 glass slides. CON, control; PFF, pulsating fluid flow. Significant effect of PFF, ** *p* < 0.01 and *** *p* < 0.001.

At different time points, live cells exposed to PFF gradually shrank towards the cell center. Cell volume decreased already significantly after 30 min PFF, and after 60 min PFF the cell volume was decreased from 5786 µm^3^ to 2774 µm^3^ (Figure 1A). Static control cells were oval-shaped with some clear lamellipodia around the cell border (Figure 1B). Nuclei of control cells were round, with a surface of rolls-and-swells (Figure 1B). PFF-treated cells contained lamellipodia as well, but generally to a lesser extend (Figure 1B). The nucleus of PFF-treated cells was ellipsoid. One hour PFF reduced cell volume by 0.3-fold (*p* < 0.0001) compared to control cells (Figure 1B). PFF reduced nucleus volume by 0.2-fold (*p* < 0.0001).

### 2.2. 2D Cell Shape and Area

**Figure 2:**
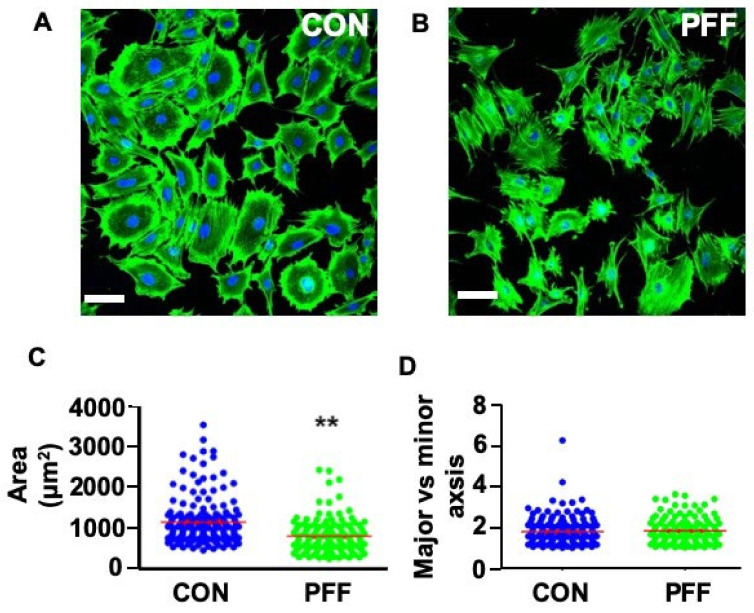
Effect of 1 h PFF on the spreading of MC3T3-E1 osteoblasts. (**A**) 2D image of fixed static control cells stained with phalloidin for F-actin (green) and DAPI for the nuclei (blue). (**B**) 2D image of 1 h PFF-treated cells stained with phalloidin and DAPI. (**C**) Cell spreading area measurement and analysis. (**D**) Cell shape/elongation measurement and analysis (cell spreading length vs. cell spreading width). *N* = 175 (control) and 165 (PFF) cells from 4 separate glass slides. CON, control; PFF, pulsating fluid flow. ** *p* < 0.01. ns, not significant. Bar = 100 µm.

Static control cells were more oval-shaped, while PFF-treated cells seemed more polygonal-shaped (Figure 2A,B). To further investigate cell spreading, cell area, length, and width were measured. PFF significantly decreased cell surface area by 0.3-fold, indicating differences in cell adhesion surface area due to PFF. The ratio of the major axis (length) versus the minor axis (width) was similar in control and PFF-treated cells (Figure 2D).

### 2.3. 3D Cell Morphology

**Figure 3:**
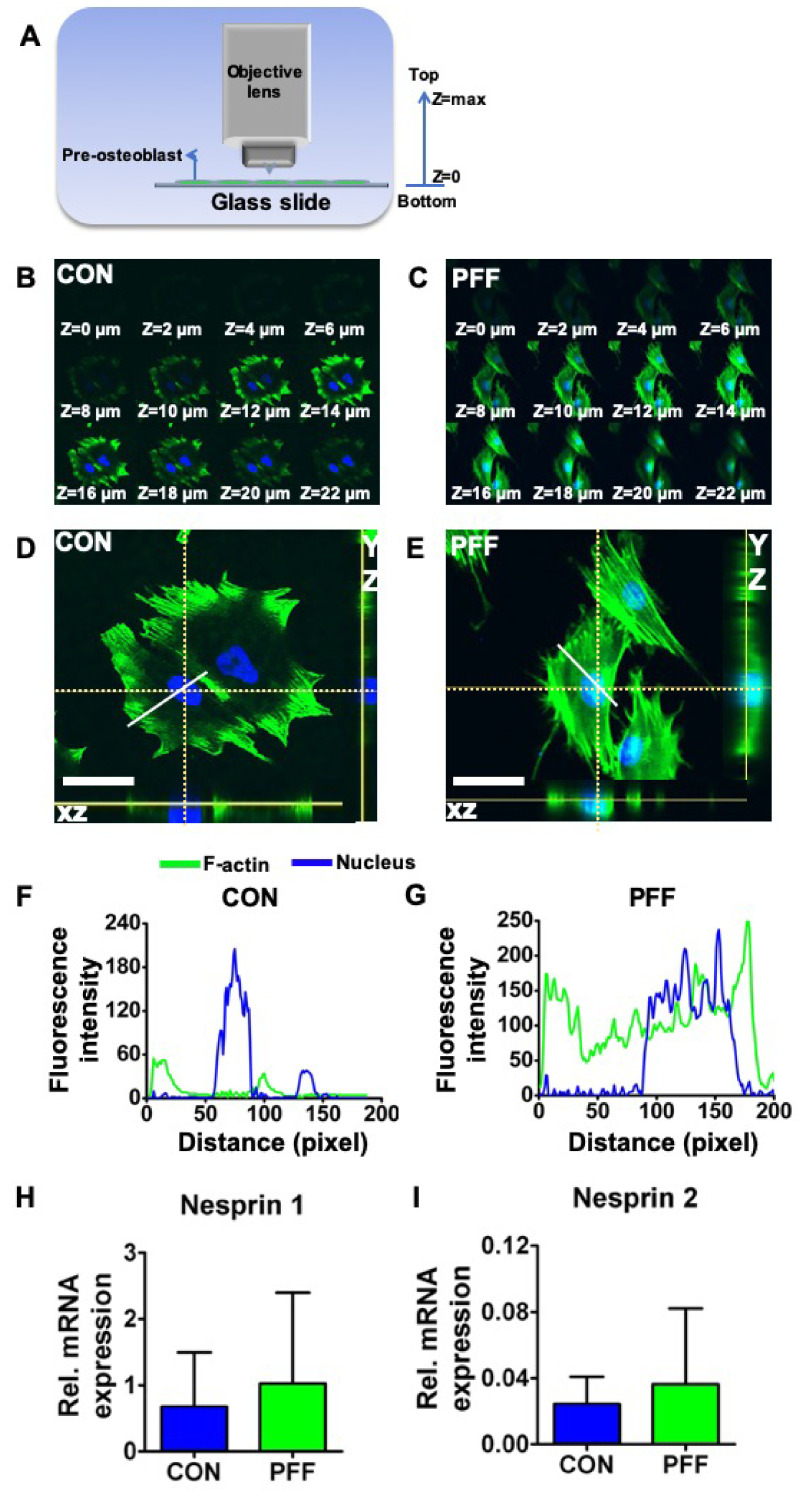
Effect of 1 h PFF on 3D F-actin distribution and nucleus position based on morphology and Z-stack analysis by laser scanning confocal microscopy (LSCM), as well as *nesprin 1* and *2* gene expression in MC3T3-E1 osteoblasts. (**A**) Illustration of confocal Z-stack scanning direction. (**B**) Z-stack scanning of a typical representative static control cell stained for F-actin and analyzed in the Z-direction at 2 µm intervals. (**C**) Z-stack scanning of a typical representative 1 h PFF-treated cell. (**D**) Top, XZ, and YZ view of a static control cell. The position of the XZ and XY view are along the dotted white lines. (**E**) Top, XZ, and YZ view of a representative cell, subjected for 1h with PFF. XZ and XY views are along the dotted white lines. (**F**) Fluorescence intensity profiles were analyzed over the solid white line drawn in Figure 3D. (**G**) Fluorescence intensity profiles over representative PFF-treated cells, detected along the solid white line visible in Figure 3E. Bar = 50 µm. (**H**) *Nesprin 1* gene expression in control and PFF-treated cells. (**I**) *Nesprin 2* gene expression in control and PFF-treated cells. CON, control; PFF, pulsating fluid flow.

The total vertical fluorescence signal span was higher (i.e., the distance over which green fluorescent signal was visible in the Z direction) in PFF-treated cells than in static control cells (Figure 3), as could be seen from the signal appearance from Z = 2 to 22 μm in the representative PFF-treated cell versus Z = 8 to 22 μm in the representative control cell (Figure 3B,C). Control cells showed green fluorescence at some distance from the nucleus in the cell periphery when viewed in Z-direction (Figure 2A, Figure 3D). In the XZ and YZ direction, little green fluorescence spots/loci were visible near the nucleus (Figure 3D). Importantly, PFF affected F-actin distribution, since green fluorescence tended to surround the entire nucleus, when cells were viewed in the Z direction (Figure 2B, Figure 3E). In the XZ and YZ direction, the results were consistent with the top view (Figure 3E). F-actin fluorescence intensity of the control cell (60 pixels, 4.9 pixels/μm) was different from that of the PFF-treated cell (100 pixels) (Figure 3F,G). *Nesprin 1* (1.52-fold, *p* = 0.61, *n* = 3) and *nesprin 2* (1.50-fold, *p* = 0.57, *n* = 3) gene expression seemed slightly (not significant) up-regulated by PFF (Figure 3H,I).

### 2.4. Expression of Paxillin

**Figure 4:**
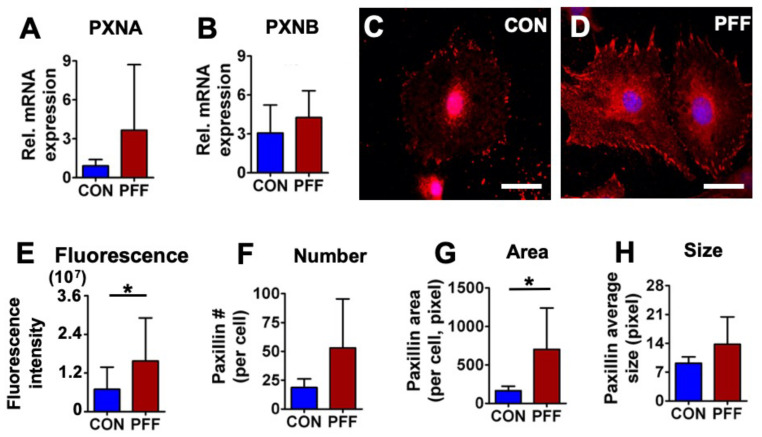
Effect of 1 h PFF on paxillin expression and distribution in MC3T3-E1 osteoblasts. (**A**) mRNA expression of *PXNA*. (**B**) mRNA expression of *PXNB*. (**C**) 2D images of control cells stained for paxillin (red) and DAPI for the nucleus (blue). (**D**) 2D images of 1 h PFF-treated cells stained for-paxillin and nucleus. Bar = 40 µm. (**E**) Fluorescence intensity of paxillin in control and PFF-treated cells. (**F**) Paxillin number in control and PFF-treated cells. (**G**) Paxillin area in control and PFF-treated cells. (**H**) Paxillin average size in control and PFF-treated cells. * *p* < 0.05. The contrast in Figure 4C and D were enhanced for clarity. CON, control; PFF, pulsating fluid flow.

To explore cytoarchitectural changes in MC3T3-E1 osteoblasts treated with 1 h PFF compared to static controls, paxillin gene and protein expression levels were measured. PFF did not enhance mRNA expression levels of *PXNA* (*p* = 0.053, *n* = 7), or *PXNB* (*p* = 0.14, *n* = 7) (Figure 4A,B). Immunofluorescent paxillin staining revealed clear clusters at the cell boundary of control cells (Figure 4C,D). PFF resulted in more paxillin dots, resembling short rods (Figure 4E–H, and Appendix A). This was confirmed by a significant 2.3-fold increase in paxillin fluorescence intensity and a 4.3-fold increase in paxillin area per cell after PFF, while paxillin number and average size remained unchanged (Figure 4).

### 2.5. Expression of Integrin-α5

**Figure 5:**
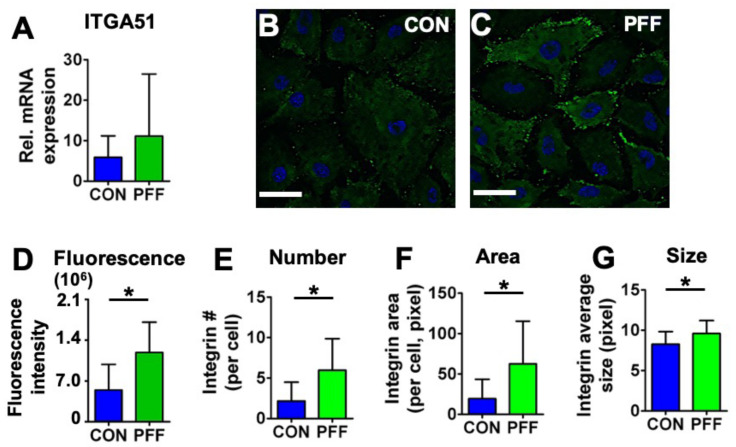
Effect of 1 h PFF on integrin-α5 expression and distribution in MC3T3-E1 osteoblasts. (**A**) mRNA expression of *ITGA51*. (**B**) 2D images of control cells stained for integrin-α5 (green) and DAPI for the nucleus (blue). (**C**) 2D images of 1 h PFF-treated cells stained for integrin-α5 (green) and nucleus (blue). Bar = 50 µm. (**D**) Fluorescence intensity of integrin-α5 in control and PFF-treated cells. (**E**) Integrin-α5 number in control and PFF-treated cells. (**F**) Integrin-α5 area in control and PFF-treated cells. (**G**) The integrin-α5 average size in control and PFF-treated cells. * *p* < 0.05. CON, control; PFF, pulsating fluid flow.

PFF did not significantly affect gene expression of *ITGA51* (Figure 5A; *p* = 0.24, *n* = 7). However, PFF enhanced the fluorescence intensity of integrin**-**α5 by 2.2-fold (Figure 5B). PFF resulted in more integrin**-**α5 dots by 2.8-fold in number per cell, 3.2-fold in area per cell, and 1.2-fold in average size (Figure 5E–G). Those changes revealed significantly more integrin-α5 clusters on the cell border of cells subjected to PFF compared to controls (Figure 5).

### 2.6. Expression of α-Tubulin and Nesprin 4

**Figure 6:**
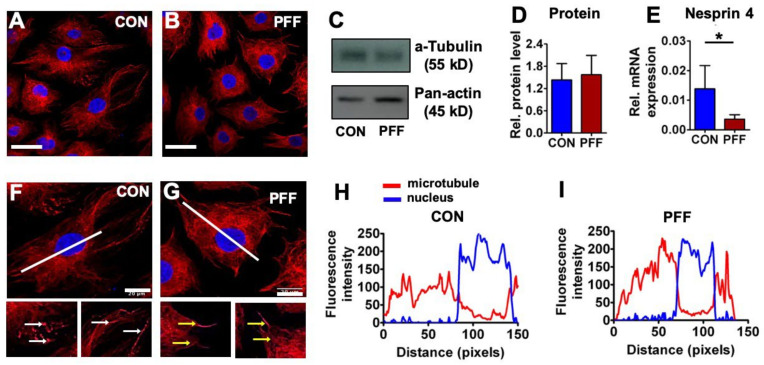
Effect of 1 h PFF on α-tubulin protein expression and distribution and *nesprin 4* gene expression in MC3T3-E1 osteoblasts. (**A**, **B**) 2D images of control cells and 1 h PFF-treated cells stained for α-tubulin (red) and DAPI for the nucleus (blue). Bar = 50 µm. (**C**, **D**, **E**) Relative α-tubulin protein expression and *nesprin 4* gene expression in control and PFF-treated cells. Pan-actin was used as a control for equal loading. (**F**, **G**, **H**, **I**) Distribution and fluorescence intensity of microtubules in MC3T3-E1 osteoblasts without or with PFF treatment. The images show the distribution of microtubules in a control and PFF-treated cell. White arrows: microtubule ends; yellow arrows: microtubule. Bar = 20 um. The graphs show the fluorescence intensity of microtubules, which correspond to the white line (from upper left to lower right) in the images. Red line: microtubule; blue line: nucleus. CON, control; PFF, pulsating fluid flow. * *p* < 0.05.

α-Tubulin immunofluorescence staining of microtubules indicated that microtubules were similarly distributed in control and PFF-treated cells, with microtubules centered around the nuclei, extending in all directions (Figure 6). The distribution of the microtubule staining intensity was similar between PFF-treated and static cultured cells, i.e., microtubule staining intensity was uniform when comparing the perinuclear area vs. the cell periphery (Figure 6). α-Tubulin protein expression levels in MC3T3-E1 osteoblasts, either or not treated with PFF, were assessed by western blot analysis (Figure 6). α-Tubulin protein expression was similar after static and PFF-treatment (Figure 6; *p* = 0.698, *n* = 4). *Nesprin 4* gene expression was significantly down-regulated by PFF treatment (Figure 6).

## 3. Discussion

This study aimed to investigate whether PFF affected cell and nucleus morphology and volume of osteoblasts, and how these effects relate to the actin cytoskeleton and focal adhesion formation. In 2D and 3D, we showed that PFF decreased cell and nucleus volume and cell surface area. These changes corresponded to increased fluorescence staining intensity for paxillin and integrin-α5, and altered distribution of F-actin and α-tubulin. This suggests that PFF alters osteoblast volume and nuclear volume, and that the changed morphology of osteoblasts after PFF coincides with changes in cytoskeletal and focal adhesion protein expression.

The actin and microtubule cytoskeleton are highly adaptive structures, rapidly adapting to internal and external triggers. Alterations in the osteoblast cytoskeletal structure in response to mechanical stimuli, such as shear stress, take place within minutes [27,28]. Hence, we have chosen this time point as an end point for our investigations. When primary mouse bone cells were exposed in vitro to different shear stresses that have been calculated to occur around osteocytes in situ in response to physiological loading, the production of nitric oxide (NO) and prostaglandin E_2_ (PGE_2_) was enhanced in a dose-dependent manner [29]. Increasing the shear stress magnitude and rate beyond physiological ranges leads to a catabolic response of MLO-Y4 osteocytes to fluid shear stress [9], more reminiscent of overloading. Here we have tested the effect of shear stress of a single magnitude within the physiological range to determine bone cell morphological changes. It was not our goal to test shear stresses resembling disuse or overuse, since these regimes are known to cause bone cell apoptosis and cell death [30]. PFF caused live cell deformation at different time points. The decreased live cell shape could result from actual cell shrinkage, but also from fluorescence bleaching. Fluorescence bleaching is affected by excited fluorophores and ambient light. Here, the cells stained for F-actin were exposed to the environment for only 1 h, and cells were kept in the dark between taking images to avoid fluorescence bleaching. In addition, fixed cells were only imaged once, and still showed a clear difference between static and PFF-exposed cells, suggesting that the decrease in cell volume was not an artifact. Cell shrinkage was not a result of cell death either (Appendix A.1). Changes in cell and nucleus volume upon mechanical loading have been shown for chondrocytes [17], and is thus, not unprecedented.

Mechanical stimuli can alter gene expression levels, and the physical connection of the cytoskeleton to the nucleus provides an optimal conduit for signal transduction from the exterior of the cell, all the way to the nucleus. Sad1 and UNC-84 (SUN), NESPRIN, and Klarsicht, ANC-1, Syne homology (KASH) proteins form a bridge across the nuclear envelope, often referred to as the linker of nucleoskeleton and cytoskeleton (LINC) complex, connecting the nucleoskeleton to the cytoskeleton [31,32,33,34,35]. In our study, we showed that nucleus volume and gene expression of nesprins were affected by PFF. Interestingly, PFF did not change the expression of actin-linked *nesprin 1* and *nesprin 2*, while the expression of microtubule-linked *nesprin 4* was down-regulated by PFF. It is a tantalizing thought that PFF-induced mechanical stimuli on the cell membrane are transferred via the cytoskeleton to the nucloskeleton via nesprins, where it mediates a change in gene expression of *nesprin 1* and *nesprin 2* in a feedback loop. However, such causal relations need to be established in future studies. 

In our study, we demonstrated that mechanical loading affects bone cell area and nucleus volume. We also found changes in gene expression for *nesprin 4*. Increasing evidence demonstrates that nuclear mechanotransduction plays a critical role in physiology and diseases, and that the response of cells to mechanical stimuli is associated with alterations of nuclear transport, unfolding or modifications of nuclear proteins, and/or changes of chromatin organization and nuclear mechanics [36,37,38]. Therefore, it is very well possible that the PFF-induced changes in bone cell and nucleus volume result in epigenetic changes.

PFF of 6.5 Pa/s shear force was used to treat the cells, since we have found earlier that the response of MC3T3-E1 pre-osteoblasts is linearly dependent on the rate of fluid shear stress, which depends on the amplitude and frequency of stress [39,40]. The fluid shear stress amplitudes and frequencies in bone have been determined by a combination of experiments and computer models, where the peak fluid shear stress around mouse osteocytes in situ has been estimated to range up to 5 Pa [28]. That this range of fluid shear stress is enough to stimulate bone cells were confirmed by in vitro studies [6,9].

**Figure 7:**
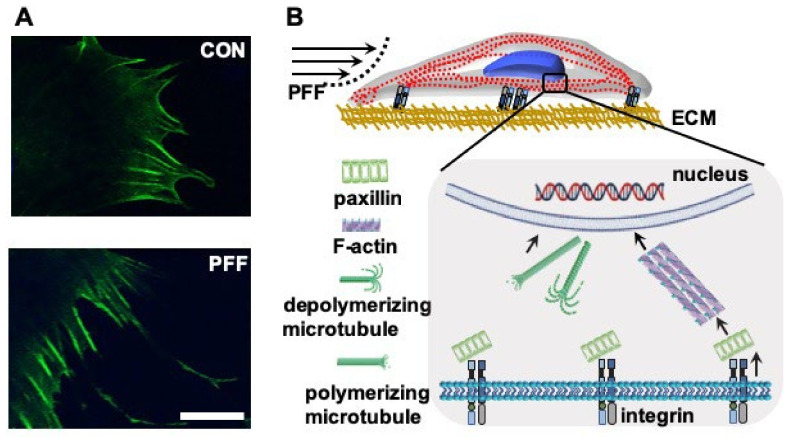
Effect of 1 h PFF on F-actin distribution at the cell boundary, and schematic summary of the changes induced by PFF in MC3T3-E1 osteoblasts. (**A**) F-actin was visualized using phalloidin. Upper image: control cell. Lower image: PFF-treated cell. Bar = 25 µm. (**B**) PFF was sensed through integrins, which formed clusters of α and β-integrins. Paxillin changed from granular to short rods. F-actin was neatly arranged, and F-actin fluorescence intensity was increased by PFF. The morphology and volume of the cell body and nucleus changed in response to PFF, which might result in changed cell function. CON, control; PFF, pulsating fluid flow.

Changes in cell and nucleus volume with PFF coincided with alterations in the F-actin network. Cells subjected to PFF clearly showed F-actin stress fibers. PFF also affected cell body shape, i.e., the cell body was more extended with a polygonal osteoblast-like shape compared to the more cuboidal control cells, as well as cell pseudopodia (Figure 7A). Our results are consistent with findings by others, also showing that during fluid shear stress-treatment of MC3T3-E1 osteoblasts, the F-actin develops into prominent stress fibers, which are oriented parallel to the cell long axis [27,41]. In addition, the tensegrity model describing the architectural basis of cells indicates that cytomembrane deformation is sensed initially as a change in the tension of F-actin stress fibers [42]. F-actin directly or indirectly interacts with the nucleus [16]. In this study, PFF affected F-actin, integrin, and microtubules, which coincided with changes in cell and nuclear area and volume. Evidence is accumulating that points to a more specific role for small heat shock proteins (sHSPs) in protecting proteins from mechanical stress. Small HSPs functions and pathways are involved in sensing and responding to mechanical cues [43]. In addition, PGE_2_ and transforming growth factor-β (TGF-β) can induce the expression of sHSPs in osteoblasts [44]. Previously we already showed that PFF stimulates PGE_2_ and TGF-β release within minutes after the start of PFF [45,46]. Therefore, it is entirely possible that also in the current study, PFF induced expression of small HSPs. In addition, evidence shows that sHSPs are involved in the protection and dynamics of the cytoskeleton [47]. For example, sHSPs can stabilize actin filaments and prevent their aggregation when the cell is treated by physical/chemical stimuli (e.g., oxidative stress, heat shock) [47]. sHSPs regulates the equilibrium between microtubules and tubulin via combining tubulin subunits [47]. 

The change in F-actin distribution in response to PFF is likely due to the rearrangement of actin fibers. In a previous study [48], we already showed that in PFF-treated osteoblastic cells, the orientation and alignment of F-actin stress fiber bundles were more homogeneous than in static control cells, with F-actin bundles running roughly parallel to the cell long axis. The connections of F-actin bundles between PFF-treated cells appeared simpler and clearer than in static control cells. The F-actin stress fiber bundles were also clearly visible above the nuclei. This data is consistent with findings by Fredrick et al., who also found that mechanical stimuli promoted the development of F-actin stress fibers, which were oriented parallel to the cell long axis in osteoblasts, whereas the F-actin stress fibers in static control cells were randomly oriented and smaller [41]. Moreover, *nesprin 1* and *2* interact with actin filaments and SUN protein, while mechanical stimuli have been shown to be transmitted from the cytoskeleton to the nuclear skeleton via SUN protein [31]. Thus, osteoblasts adapt to external influences, such as mechanical stimuli by changes in F-actin, which affects cell morphology and function, and possibly affected nucleus volume (Figure 7B). The morphological changes did not hold once the force was removed. We observed that cell morphological changes returned to normal in PFF-treated cells at 4 h after removal of PFF, as PFF-treated cells showed similar morphological features as static control cells at this time point (Appendix A.2). Mechanical loading-induced changes in cell and nucleus morphology may drive signaling pathways affecting osteogenic differentiation and bone cell function [49]. Future studies should investigate how long the morphological changes of PFF-treated cells return to normal after removal of mechanical loading, and the relationship of morphological changes and cell function.

The osteoblastic stress response to mechanical stimulation likely involves microtubules that contain α-microtubulin and β-microtubulin, which make up the structural core [50]. Microtubule depolymerization and polymerization in intact cells depend on whether cells are in homeostasis or unstable, moving, in the mitotic stage, or whether they are subjected to external stimuli, e.g., mechanical loading [50,51]. Moreover, microtubules switch quickly between being stably growing and rapidly shrinking [52], which allows them to adapt [52]. In this study, immunofluorescence followed by LSCM revealed that PFF affected microtubule distribution and fluorescence intensity. *Nesprin 4* gene expression was decreased by PFF. *Nesprin 4* is known to interact with microtubules and SUN protein [31]. Mechanical loading of osteoblasts, thus, likely caused rapid depolymerization and repolymerization of microtubules. PFF altered both actin filaments and microtubules, which likely contributed to changes in cell and nucleus volume. The sequence of PFF-induced changes in polymerization and depolymerization of both microtubules and actin filaments, and the exact impact of these cytoskeletal changes on cell morphology remain to be determined (Figure 5B).

Both paxillin and integrin staining intensity was stronger after PFF-treatment. When paxillin expression is up-regulated in embryonic stem cells, the cell shape turns less round [53]. The currently observed changes in nucleus volume may, thus, be related to alterations in paxillin. In our study, the focal adhesion (paxillin) area was increased by PFF. This data suggests that focal adhesions are indeed important for PFF-induced mechanotransduction in osteoblasts, as has been reported by Young and colleagues [54]. Interestingly, soft stiffness substrates promote the appearance of osteocyte-line features in MC3T3-E1 cells seeded at low density [55]. Soft substrates are generally associated with smaller focal adhesion size. On the other hand, substrates of higher stiffness enhance Rho activity, F-actin formation, focal adhesion size, and osteogenic differentiation of mesenchymal stem cells (MSCs) compared to less stiff substrates [56]. It is this likely that the increased focal adhesions will affect the differentiation of the osteoblasts, but to what extend is it unclear. We have used paxillin as a measure for focal adhesion size. The cytoplasmic domain of the beta-subunit of integrins (e.g., α5β1) interacts directly with talin, and in turn, talin interacts with both vinculin and paxillin. Focal adhesion kinase (FAK) localizes to focal adhesions because it binds to paxillin [57]. Talin and paxillin may, thus, be more “direct” measures of focal adhesion size than FAK. On the other hand, paxillin and FAK, but not the linker protein talin, actively regulate extra cellular matrix (ECM)-actin linkage through modifications, such as phosphorylation or enzymatic cleavage of proteins that comprise the link. Paxillin, thus, acts as both a linker protein and a mediator of molecular processes, which talin and FAK do not. In addition, paxillin has been associated with the sensing of fluid shear stress in a multitude of cell types, including osteocytes [58]. Osteoblasts appeared to produce more integrin-α5 clusters after PFF. This data is consistent with data from others showing that integrin-α5 gene expression is slightly increased by mechanical loading in human tendon stem/progenitor cells. The mechanical loading-induced changes in actin filaments, paxillin, integrins, microtubulin, and nucleus might directly or indirectly be related to PFF-induced alterations in osteoblast function, such as matrix formation. Moreover, we found that MC3T3-E1 osteoblasts exposed to 1 h PFF enhanced collagen formation at three weeks post-PFF, but alkaline phosphatase (ALP) activity and the formation of mineralized nodules was unchanged (Appendix A.3). One hour PFF treatment transiently changed bone cell morphology in for up to 4 h after PFF, and collagen production three weeks later. PFF set into motion a cascade of events, possibly starting with a rearrangement of cytoskeletal fibers and altered nucleus volume. We can speculate that the mechanical stimulus was transduced via nesprins all the way to the nucleus, where transcription of early transcription factors, such as runx2 and osterix may have been up-regulated, which in turn enable transcription of bone matrix genes. Although we did not delineate the sequence of events in the current manuscript, the cascade of events resulted in elevated collagen production up to 3 weeks later. Others have found increased differentiation of MC3T3-E1 osteoblasts several weeks after a single bout of loading is associated with induction of bone morphogenetic protein 2 expression, which then stimulates differentiation [59].

PFF decreased osteoblast cell and nucleus volume. Changes in bone cell nucleus morphology are known to affect gene expression of nuclear matrix and cytoskeletal components in cells cultured on microfabricated substrates [60]. Gene expression in T-cells is also modulated by nuclear shape [61]. It has been shown that changes in bone cell nucleus morphology affect gene expression of nuclear matrix and cytoskeletal components in cells cultured on microfabricated substrates [60]. Therefore, the observed nucleus volume in our study may also have affected gene expression and cell function, although specialized studies further focusing on the relation between nucleus volume and cell behavior are needed to substantiate such a finding. Moreover, the position of genes with respect to the nuclear envelope affects the regulation of their expression [60,62,63]. In our study, nucleus deformation might have affected gene expression and cell function. Although no significant differences were found in gene expression of paxillin and integrin-α5 after PFF-treatment, *nesprin 4* expression was significantly altered. However, it has been shown previously that expression of amongst others insulin-like growth factor-I (IGF-I), vinculin (VCL), and anti-polyclonal (APC) are affected after 1 h PFF-treatment of MC3T3-E1 cells [64,65,66]. We demonstrated that PFF affects F-actin, integrin, microtubule, cell area, and cell and nucleus volume. Others have shown that Rho GTPases are molecular switches that control various signal transduction pathways in all eukaryotic cells. They are known principally for their pivotal role in regulating the actin cytoskeleton, but their ability to influence cell polarity, microtubule dynamics, membrane transport pathways, and transcription factor activity is probably just as significant [67]. Therefore, it may be hypothesized that PFF changed Rho GTPases, resulting in changes in the actin cytoskeleton in MC3T3-E1 pre-osteoblasts. The Rho GTPases encompass Cdc42, Rac1, and ras homolog family member A (RhoA), of which Rho activation is known to stimulate F-actin fiber formation and focal adhesion formation [68], as we observed after PFF. We did not observe cytoskeletal changes consistent with Rac1 and Cdc42 activation. It is, thus, tantalizing to assume that PFF enhances RhoA activity. We have previously performed RhoA activity measurement (quantified using G-LISA™ Small G-protein Activation Assay) in PFF-treated and static human osteoblasts (cultured cells derived from human collagenase-treated bone pieces, three separate donors). RhoA activity was nearly abolished in response to treatment with the Rho inhibitor Y27632, demonstrating the efficiency of the assay. To our great surprise, though, PFF for 1h, comparable in magnitude and frequency to the stimulation in the current manuscript, reduced RhoA activity by 2 to 3-fold (unpublished results). In other words, PFF strongly affects RhoA activity, but either activation of RhoA is transient, and has passed its peak at 1h post-PFF, or some other factor (e.g., small HSPs) is responsible for the actin redistribution observed after PFF. Future studies should explore the mechanisms behind the effect of PFF on nuclear volume, cytoskeletal and cell volume changes in more detail, and try to find a causal relation between PFF-induced changes in cell and nucleus volume and cell behavior. 

## 4. Materials and Methods

### 4.1. Cell Culture

MC3T3-E1 osteoblasts (passage 16–28) were cultured in α-minimal essential medium (α-MEM; Gibco, Paisly, UK) with 10% fetal bovine serum (FBS; Gibco), 300 μg/mL penicillin (Sigma-Aldrich, St. Louis, MO, USA), 250 μg/mL streptomycin (Sigma-Aldrich), and 1.25 μg/mL fungizone (Gibco) in a humidified atmosphere of 5% CO_2_ in air at 37°C. Upon reaching 80−90% confluence, cells were harvested with 0.25% trypsin and 0.5 mM EDTA for 5 min.

### 4.2. Treatment with Pulsating Fluid Flow

One day before PFF-treatment, MC3T3-E1 osteoblasts were harvested and seeded at 1×10^3^ cells/cm^2^ (for cell structure analysis), and at 3 × 10^3^ cells/cm^2^ (for gene and protein expression analysis) on poly-L-lysine-coated (50 µg/mL; poly-L-lysine hydrobromide; Sigma-Aldrich) glass slides (24 × 24 × 0.15 mm or 36 × 76 × 1 mm). Prior to 1 h PFF, the medium was refreshed by α-MEM containing 2% FBS and antibiotics. PFF was generated using a flow apparatus containing a parallel-plate flow chamber [69]. Two different chambers were used, i.e., a “big chamber” (5.8 × 3.2 × 0.03 cm (inner dimension)) for measuring gene and protein expression, and a “small chamber” (1.4 × 1.4 × 0.02 cm (inner dimension)) for measuring cell morphology and volume. In both chambers, cells were treated with the same intensity of PFF (amplitude: 1.0 Pa, peak shear stress rate: 6.5 Pa/s, frequency: 1 Hz) for 1 h at 37 °C (for fixed cell analysis) or at room temperature (for live cell analysis). Static control cultures were kept in a petri-dish under similar conditions as experimental cultures.

### 4.3. Cell Morphology

After 1 h PFF, MC3T3-E1 osteoblasts were fixed with 4% paraformaldehyde solution at 37 °C for 15 min, treated with 0.2% TritonX−100 (Sigma-Aldrich) for 15 min, and blocked in 5% bovine serum albumin (BSA) for 30 min. Expression of F-actin, phospho-paxillin (p-paxillin), α-tubulin, and integrin-α5 was analyzed by immunofluorescence staining with respectively rhodamine-phalloidin (Invitrogen, Fisher Scientific, Carlsbad, CA, USA), p-paxillin pTy31 polyclonal rabbit immunoglobulin G (IgG) (ab32084, Abcam, Cambridgeshire, UK), α-tubulin (Abcam), and α5 rat monoclonal IgG−2a (Abcam). The secondary antibody was Alexa Fluor−488 goat anti-rat IgG (Abcam), or Alexa Fluor−555 goat anti-rat IgG (Abcam). Nuclei were stained with 1 µg/mL DAPI (Sigma-Aldrich). After glycerol mounting, cell imaging was performed by laser scanning confocal microscopy (LSCM; Nikon, A1R/A1, Tokyo, Japan). LSCM images were captured randomly at three representative sites/glass slide at 40x magnification. Captured images did not overlap. The LSCM z-stack was executed every 0.15 µm in a whole cell. Fluorescence intensity (the value of Integrated Density. Because osteoblasts are flat, raw Integrated Density might have a similar level of fluorescence calculated by CTCF (Corrected total cell fluorescence = Integrated Density—(Area of selected cell × Mean fluorescence of background readings)) as measured by Image J software (https://imagej.net/Downloads). For F-actin-stained cells, cell boundaries were drawn using the toolbar, and cell area, length, and width were determined. F-actin fluorescence in the Z direction was visualized via Image J software. Paxillin and integrin-α5 number, area, and average size were also determined using Image J software (image type: 8-bit; threshold: 40/255; size: 5-infinity; circularity: 0.00−1.00). Fifty-two (control) and 47 (PFF) cells from six glass slides from 3 independent experiments (*n* = 3) were measured.

To observe the live cell morphology before and after PFF, MC3T3-E1 osteoblasts seeded on poly-L-lysine coated glass slides were stained by SiR-actin (Spirochrome, Stein-am-Rhein, Switzerland) for F-actin in a culture medium with 10% FBS for 4 h before the start of PFF. Live cell images were captured by SP8 confocal microscopy (Leica, Solms, Germany) at 0, 10, 20, 30, 40, 50, and 60 min after the start of PFF. Four cells were imaged per glass slide. In total, six glass slides from six independent experiments were analyzed.

### 4.4. Cell and Nucleus Volume

LSCM images were used to select the target cell by utilizing Image J software. Cell threshold data were set to clearly display the cell boundary to reconstruct cell and nucleus shape and to calculate their volume. Afterward, cell and nucleus volume were quantified by MeasureStack software (http://www.optinav.info/MeasureStack.htm). MITK software was utilized to reconstruct the 3D-shape of the cell and nucleus (http://www.mitk.net/download_mitk_ch1.html). The fold-change in cell and nuclear volume was quantified by this equation (mean volume _CON_ - mean volume _PFF_)/mean volume _CON_).

### 4.5. RNA Isolation and Quantitative Real Time Polymerase Chain Reaction (RT-PCR)

Total RNA was isolated from MC3T3-E1 osteoblasts using 700 µl Trizol (Invitrogen, Carlsbad, CA, USA). Complementary DNA synthesis was performed using 750 ng of total RNA according to the First Strand cDNA Synthesis kit (Thermo Fisher Scientific, Vilnius, Lithuania) in a thermocycler GeneAmp^®^ PCR System 9700 PE (Applied Biosystems, Foster City, CA, USA). cDNA was diluted to a final concentration of 2 ng/µL for gene expression analysis. RT-PCR reactions were performed using 4 µl cDNA per reaction, 0.5 µl forward-primer (1 µM), 0.5 µl reverse-primer (1 µM), and 5 µl LightCycler^®^ 480 SYBR^®^ Green I Mastermix (Roche Diagnostics, Mannheim, Germany) in a LightCycler^®^ 480 (Roche Diagnostics, Mannheim, Germany). RT-PCR conditions for all genes were as follows: 10 min pre-incubation at 95°C, followed by 45 cycles of amplification at 95°C for 10 s, 56 °C for 5 s, 72 °C for 10 s, and 78 °C for 5 s, after which melting curve analysis was performed. With LightCycler^®^ software (version 1.2), crossing points were plotted versus the serial dilution of known concentrations of the internal standard. Values of target gene expression were normalized to *PBGD* (Forward: AGTGATGAAAGATGGGCAACT; Reverse: TCTGGACCATCTTCTTGCTGA) to obtain relative gene expression. RT-PCR was used to assess expression of the following genes: paxillin-a (*PXNA;* Forward: CAGTCCGCAGCGAGTCA; Reverse: CCTGGGCCATGAACTTGAAATC), paxillin-b (*PXNB;* Forward: ACCAGGGAGAGATGAGCAGT; Reverse: AGGCCCTGCATCTTGAAATCT), integrin-α5 (*ITGA51;* Forward: GGAAGGGACGGAGTCAGTG; Reverse: TAGACAGCACCACCTTGCAG), *nesprin 1* (mNSP1var23; Forward: CTGCTGCTTATTGGACTCACCT; Reverse: GAGGAGGACCGTTGGTATATCTG), *nesprin 2* (mNSP2; Forward: GAGACGCTCCTTCCTCTCAA; Reverse: TTGTCAAGGCAAAGTCACTCC), and *nesprin 4* (mNSP4; Forward: TGCACCTGAGGAAGAGACAA; Reverse: CCGGAAGTTCAACCTCAACA). Data were obtained from 14 glass slides from 7 separate experiments (*n* = 7 for paxillin and integrin, *n* = 3 for *nesprin 1, 2*, and *4*).

### 4.6. Protein Isolation and Western Blot

MC3T3-E1 osteoblasts were lysed in 600 µL isopropyl alcohol (IPA) buffer (Sigma-Aldrich). Total protein was measured using Pierce^TM^ BCA Protein Assay Kit (Thermo Fisher Scientific). Samples were separated by 10% sodium dodecyl sulfate-polyacrylamide gel electrophoresis (SDS-PAGE) gels. Blots were blocked in 5% BSA for 1 h, followed by incubation overnight at 4°C with primary antibodies against α-tubulin (Abcam) and pan-actin (Abcam). Then 1 h incubation with goat anti-rabbit IgG horseradish peroxidase (HRP) (Abcam) conjugated with horseradish peroxidase was performed, and membranes were monitored with a western light chemiluminescent detection system (Thermo Fisher Scientific). Protein bands were analyzed and quantified with Image J, and normalized to standard pan-actin levels. Data were obtained from eight glass slides from four separate experiments (*n* = 4).

### 4.7. Statistical Analysis

All data are presented as mean ± standard deviation (SD). Data were statistically tested with a t-test, and considered significant if *p* < 0.05. Statistical analysis was performed using International Business Machines Corporation (IBM^®^) Statistical Product and Service Solutions (SPSS^®^) Statistics version 21 software package (SPSS Inc., Chicago, IL, USA).

## 5. Conclusions

In this article, we demonstrated that PFF induces substantial changes in osteoblast cell and nucleus morphology and volume, which are accompanied by altered spatial and temporal distribution of adhesion and cytoskeletal proteins. More insights into the mechanisms driving morphological changes in bone cells, and thereby, possibly bone cell behavior, in response to mechanical cues, will aid the guidance of clinical treatment, particularly in the field of orthodontics, (oral) implantology, and orthopedics.

## Figures and Tables

**Figure A1:**
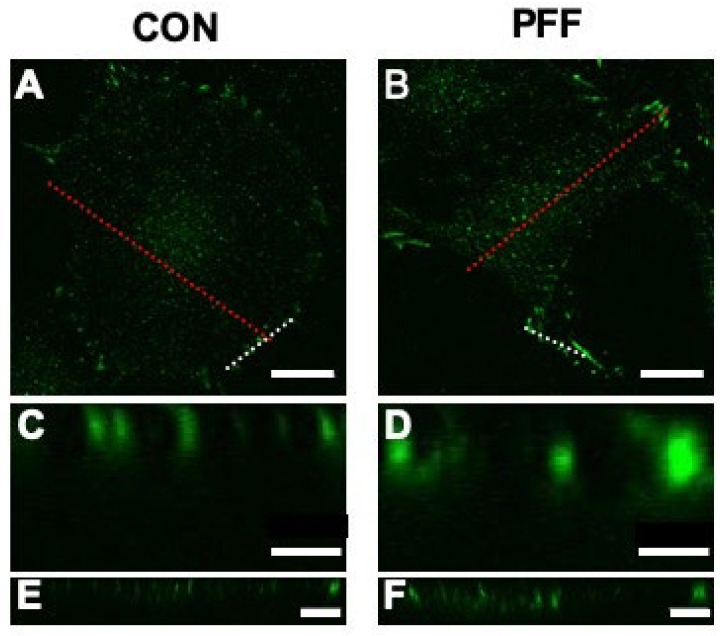
Focal adhesions visualized by staining of paxillin in MC3T3-E1 cells cultured without or with 1 h PFF. Line tool (Image J) was used to draw the target area. The “Reslice” function was used, and the following parameters applied: Output spacing (microns): 0.125, Slice count: 1, Avoid interpolation (use 1 pixel spacing). (**A**) Paxillin morphology in control cells without PFF. Bar: 20 μm. (**B**) Paxillin morphology in PFF-treated cell. Bar: 20 μm. (**C**) Side view of the paxillin staining based on the white dotted line in (A). Bar = 10 μm. (**D**) Side view of paxillin based on the white dotted line in (B). Bar: 10 μm. (**E**) Side view of the paxillin staining based on the red dotted line in (A). Bar: 5 μm. (**F**) Side view of the paxillin staining based on the red dotted line in (B). Bar: 5 μm. CON, control; PFF, pulsating fluid flow.

**Figure A2:**
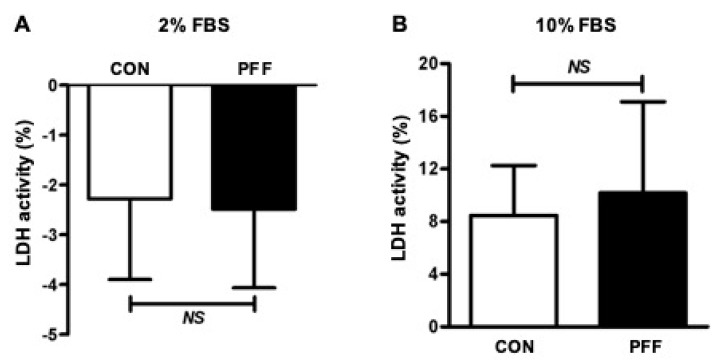
Lack of cytotoxic effect of different culture media before and after PFF on MC3T3-E1 osteoblasts as measured by lactate dehydrogenase activity. (**A**) Before PFF treatment, LDH activity of MC3T3-E1 osteoblasts in α-MEM containing 2% FBS for 1 h; (**B**) After PFF treatment, LDH activity of MC3T3-E1 osteoblasts without and with PFF in α-MEM containing 10% FBS for 1 h. NS: No significance. LDH, lactate dehydrogenase; FBS, fetal bovine serum; CON, control; PFF, pulsating fluid flow.

**Figure A3:**
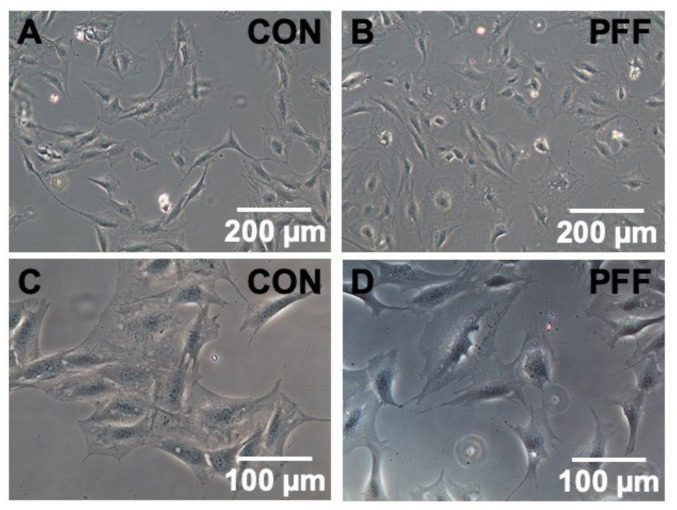
Cell morphology of MC3T3-E1 pre-osteoblasts at 4 h post-incubation without loading, following treatment with or without 1 h PFF. (**A**,**C**) Static control cells. (**B**,**D**) 1 h PFF-treated cells.

**Figure A4:**
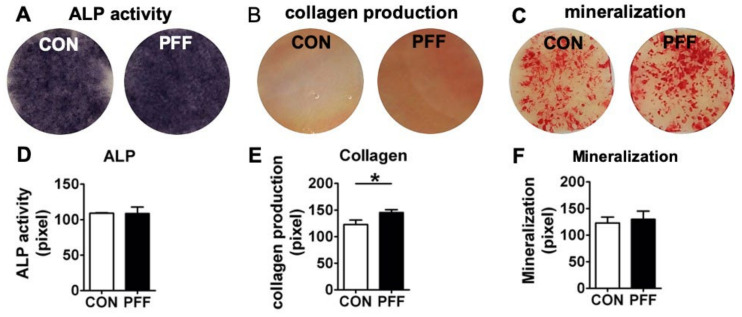
Effect of PFF on osteogenic differentiation of MC3T3-E1 osteoblasts. Prior to 1 h PFF, the medium was refreshed by α-MEM containing 2% FBS and antibiotics. PFF (amplitude: 1.0 Pa, peak shear stress rate: 6.5 Pa/s, frequency: 1 Hz) was generated for 1h using a pump and a parallel-plate flow chamber. Static control cultures were kept in a petri-dish under similar conditions as experimental cultures. Cells were post-incubated for up to 28 days. (**A**,**B**,**C**) The optical images show alkaline phosphatase (ALP) activity, collagen production, and mineralization after 21 or 28 days of cell culture. (**D**,**E**,**F**) Quantification of ALP activity, collagen production, and mineralization after 21 or 28 days of cell culture. CON, control; PFF, pulsating fluid flow. * *p* < 0.05.

## Abbreviations

ALP	Alkaline phosphatase
APC	Anti-polyclonal
α-MEM	α-Minimal essential medium
BCA	Bicinchoninic acid
BSA	Bovine serum albumin
cDNA	Complementary DNA
CON	Control
CSC	China Scholarship Council
CTCF	Corrected total cell fluorescence
DAPI	4′,6-Diamino−2-phenylindole
2D	Two dimensional
3D	Three dimensional
ECM	Extracellular matrix
EDTA	Ethylemediaminetetraacetic acid
FAK	Focal adhesion kinase
FBS	Fetal bovine serum
HRP	Horseradish peroxidase
IBM	International Business Machines Corporation
IGF-I	Insulin-like growth factor−1
IgG	Immunoglobulin G
IPA	Isopropyl alcohol
*ITGA51*	Integrin-α5
KASH	Klarsicht, ANC-1, Syne homology
LDH	Lactate dehydrogenase
LINC	Linker of nucleoskeleton and cytoskeleton
LSCM	Laser scanning confocal microscope
MC3T3-E1	Mouse calvaria 3T3-E1
MITK	Medical imaging interaction toolkit
MSCs	Mesenchymal stem cells
NO	Nitric oxide
p-actin	Pan-actin
*PBGD*	Porphobilinogen deaminase
PFF	Pulsating fluid flow
PGE_2_	Prostaglandin E_2_
p-Paxillin	Phospho-paxillin
*PXNA*	Paxillin-a
*PXNB*	Paxillin-b
RhoA	Ras homolog family member A
RT-PCR	Real time polymerase chain reaction
SD	Standard deviation
SDS-PAGE	Sodium dodecyl sulfate-polyacrylamide gel electrophoresis
sHSPs	Small heat shock proteins
SiR	Spirochrome
SPSS	Statistical Product and Service Solutions
SUN	Sad1 and UNC-84
TGF-β	Transforming growth factor-β
VCL	Vinculin

## Appendix A

### Appendix A.1

A potential cytotoxic effect of different culture media before and after PFF on MC3T3-E1 osteoblasts was assessed by measuring lactate dehydrogenase (LDH; Roche, Mannheim, Germany). Prior to 1 h PFF treatment, the medium of MC3T3-E1 osteoblast cultures was refreshed by α-MEM containing 2% FBS, 300 μg/mL penicillin, 250 μg/mL streptomycin, and 1.25 μg/mL fungizone. After 1 h of culture, the medium was collected and replaced by α-MEM containing 10% FBS, 0.1% penicillin, 0.1% streptomycin, and 0.5% fungizone. After 1 h with or without PFF treatment, the medium was also collected. The medium of all samples (100 µl) were pipetted into 96-well plates and incubated with 100 µl reaction mixture for 20 min at room temperature in the dark. The LDH activity was measured in a Synergy HT^®^ spectrophotometer (BioTek Instruments, Winooski, VT, USA) at 490 nm.
